# 1029. Torus Synestia Nucleic Acid Analysis Platform for Fast, High Multiplex Analysis of Nucleic Acids With Single-Nucleotide Discrimination Level

**DOI:** 10.1093/ofid/ofab466.1223

**Published:** 2021-12-04

**Authors:** Tyler Rockwood, Andrew Sullivan, Jahnavi Gandhi, Sarah Gruszka, Brian Turczyk, Dmitriy Khodakov

**Affiliations:** TORUS BIOSYSTEMS, INC., Cambridge, Massachusetts

## Abstract

**Background:**

Nucleic acid amplification testing (NAAT) is an essential tool both for biomedical research and for clinical molecular diagnostics. Currently, there are multiple NAAT platforms available, each offering certain performance and utility advantages and disadvantages as compared to each other. Next generation NAAT platforms aim to deliver increased target detection sensitivity and specificity, low limits of target detection, quantitative high multiplex target capacity, rapid time to results, and simple sample-to-answer workflow.

**Methods:**

Here we describe the Torus Synestia System, a NAAT platform capable of rapid, highly multiplexed amplification and detection of both DNA and RNA targets. The platform comprises a small, portable (~ 2kg) amplification and detection device and a disposable single-use cartridge housing a PCR amplification chamber with an integrated label-free microarray for real-time data acquisition and interpretation. The platform offers a 30-min turnaround time with a detection limit of 10 DNA/RNA molecules per assay and single nucleotide discrimination.

**Results:**

We demonstrate the Synestia System performance and utility with three distinct molecular applications: 1) detection of 20 genetic loci and 30 single nucleotide polymorphisms in human genomic DNA; 2) detection and genotyping of 43 unique bacterial species associated with human urinary tract infections; and 3) detection and profiling human respiratory viral pathogens including SARS-CoV-1/2, seasonal coronaviruses, Influenza A/B, and human respiratory syncytial viruses. In addition, the single-nucleotide specificity of our label-free microarray probes allowed for robust identification and discrimination of newly emerging SARS-CoV-2 lineages, such as B.1.1.7 (a.k.a. UK), B.1.351 (a.k.a. South African), P.1 (a.k.a. Brazilian), and B.1.617 (a.k.a. Indian).

**Conclusion:**

The Torus Synestia System has broad applicability in both clinical and research environments. We are confident that the Torus Synestia System will revolutionize syndromic diagnostics at the point of care (PoC) and lead to improved response times during future epidemic and pandemic pathogen outbreaks.

**Disclosures:**

**All Authors**: No reported disclosures

